# Fingerprinting of Plum (*Prunus domestica*) Genotypes in Lithuania Using SSR Markers

**DOI:** 10.3390/plants12071538

**Published:** 2023-04-03

**Authors:** Raminta Antanynienė, Jūratė Bronė Šikšnianienė, Vidmantas Stanys, Birutė Frercks

**Affiliations:** Lithuanian Research Centre for Agriculture and Forestry, Institute of Horticulture, Department of Orchard Plant Genetics and Biotechnology, Kaunas District, LT-54333 Babtai, Lithuania

**Keywords:** fingerprinting, *Prunus domestica*, microsatellites, genetic diversity

## Abstract

This study’s aim was to evaluate the genetic diversity of European plum (*Prunus domestica*) cultivars and hybrids in Lithuania using SSR markers. In total, 107 plum genotypes (including 68 European plum cultivars and 39 hybrids) from the genetic resources collection of the Institute of Horticulture of the Lithuanian Research Centre for Agriculture and Forestry (LRCAF IH) were evaluated using nine microsatellite markers (SSRs) previously published and suggested by the European Cooperative Programme for Plant Genetic Resources (ECPGR). Up to six alleles per locus with each primer pair were generated for some genotypes due to the hexaploidy of plums. The number of alleles in each primer ranged from 18 to 30, with an average of 24.33. The highest number of alleles was generated with the PacA33 primer pair (30). The most informative primer, according to the PIC value, was BPPCT007. Sixty-two unique alleles (representing 39.5% of all polymorphic alleles) have been detected in the plum germplasm developed in Lithuania. According to UPGMA cluster analysis, 58 European plum genotypes were separated into eight groups without any relation to fruit color or shape. By genetic diversity (UPGMA) and structure (Bayesian) analysis, European plum hybrids were grouped into clusters according to their pedigree.

## 1. Introduction

A member of the *Rosaceae* family, genus *Prunus*, the European plum (*Prunus domestica* L.) is grown in temperate zones for its fleshy fruit, consumed fresh, dried, or canned worldwide [1]. The European plum originated in southeastern Europe and western Asia. In total, about 6000 plum cultivars that are counted worldwide belong to 19–40 plant species [2]. The European Cooperative Program for Plant Genetic Resources (ECPGR) aims to ensure the long-term conservation of *Prunus* germplasm, including *Prunus domestica,* in Europe [3].

Plum cultivars have different pomological characteristics and significant variability in phenotypic values within the germplasm. The precise origin of *Prunus domestica* has been known for only a century. Crane and Lawrence [4] performed the breeding experiment of the European plum (2n = 6x = 48), analyzing that the European plum is an interspecific hybrid of diploid *P. cerasifera* and tetraploid *P. spinosa.* This experiment was confirmed by molecular biology methods [5].

In plant breeding, it is crucial to know the parental form of the species and the phenotypic and genetic characteristics to ensure that the correct germplasm or gene of interest is included in modern plant breeding programs [6]. Modern methods in plant breeding have been used to adapt plants to various climatic changes and make them resistant to abiotic (winter hardiness, temperature fluctuations, heating) and biotic factors (e.g., Sharka disease, bacterial canker, brown rot) [7]. It is essential to maintain unique cultivars with valuable traits and well-documented cultivars throughout Europe to build a decentralized European germplasm collection and evaluate the variability of European plum cultivars in different geographical locations [3]. The breeding of the European plum in Lithuania is relatively young, starting in 1952. The breeding tasks were to produce new cultivars resistant to winter harshness, fungal and other diseases, with good yield and quality [8,9]. Using traditional breeding methods, 133 cross-combinations involving 59 plum cultivars have been conducted in Lithuania to develop new cultivars [9].

It is important to analyze European plum cultivars growing in Lithuania and newly introduced hybrids through morphological, phenological and molecular characterizations [10]. In the last century, local plum cultivars have changed due to climate conditions. The crucial step to conserve biodiversity in crops is the analysis of genetic variation in germplasm collections [11]. Molecular biology methods, such as RAPD [12], RFLP [11,13,14], ISSR [15] and SSR [1,3,5,6,10,11,16,17,18,19,20,21,22,23,24,25,26,27,28] were used for genotyping *Prunus domestica* species at the genomic level. *Prunus domestica* is a less-analyzed specie of the *Prunoideae* subfamily due to its polyploidy [16]. Analysis of *Prunus domestic* molecular markers is complicated due to sequencing, assembling genomes and analyzing polyploid genomic data [29]. For this reason, the SSR method for genetic fingerprinting was adopted later in European plum than in diploid *Prunus* species, such as peach or sweet cherry [3]. However, genetic markers, such as SSR, should disclose multiple alleles and easily separate genetically similar individuals and homologous genomes [16].

The ECPGR confirmed a set of nine SSR markers for SSR analysis of *Prunus domestica* [3]. Since then, it has been used in several studies for genetic diversity analysis of plums worldwide [6,23,25]. A standardized SSR primer set allows the comparison of results between different countries and laboratories [3]. European plum cultivars in Lithuania have not been studied with any molecular markers. Therefore, this study aimed to identify European plum cultivars and hybrids developed in Lithuania using SSR markers and evaluate the genetic diversity of the Lithuanian plum germplasm.

## 2. Results

A total of 68 European plum cultivars (including six foreign reference cultivars (R-Plum group) and 14 cultivars of Lithuanian-origin (LT origin-Plum group), 48 accessions from Lithuania genetic resources collection of plum (LT-Plum group) and 39 hybrids were analyzed using nine microsatellite loci published by Nybom et al. [3]. In total, 219 polymorphic alleles were identified by evaluating primer informativeness for cultivars and hybrids. The number of alleles in all analyzed cultivars (R-plum, LT origin-Plum group and LT-Plum groups) and hybrids varied from 18 to 30 per marker, with an average of 24.33 (Table A1). The most informative markers with the highest number of alleles (30) were locus PacA33. The lowest number of polymorphic alleles (14) was amplified with the BPPCT014 locus. Molecular profiles for all European plum cultivars are presented in Table S1.

According to the evaluation of plum cultivars, the total number of polymorphic alleles in reference cultivars (R-Plum) was smaller (95 alleles) compared to cultivars of Lithuanian origin (LT origin-Plum group) (141 alleles) (Table 1). The number of alleles in the R-Plum group ranged from 6 to 13 (an average of 10.56), with the highest number of alleles (13) observed at loci BPPCT034, BPPCT039, and CPSCT026. The lowest number of alleles was generated by the UDP98-407 locus (6). The range of the allele number in the LT origin-Plum group was wider (from 11 to 21, with an average of 15.67). The highest number of alleles was found in BPPCT040 (21) and the lowest number in UDP98-407 (11). In the LT origin-Plum group, the informativeness of SSR primers according to allele number is higher than in the R-Plum group.

The maximum number of alleles in genotypes of both groups was in the range of 3–6. Among all analyzed cultivars, the lowest number of alleles in genotype (3) was observed in locus UDP98-407. In referenced cultivars (R-Plum group), the lowest number of alleles in the genotype was in loci UDP98-407 and CPSCT026 (Table 1 and Table S1).

In both groups of cultivars, the allele size ranged from 96 to 258 bp, with the smallest size in primer UDP96-005 and the highest in primer BPPCT034, respectively (Table 1). In loci BPPCT034 and BPPCT014, the allele size range was the same in both groups. The allele size range differed between cultivar groups in the remaining 7 SSR loci. Despite the lower number of analyzed samples in the R-Plum group, the allelic size range for BPPCT039, UDP98-407, and PacA33 loci is more extensive than in the LT origin-Plum group. In BPPCT040, BPPCT007, CPSCT026 and UDP96-005 loci, the allele size range is more extensive in the LT origin-Plum group.

Observed heterozygosity (H_o_) was high, ranging from 0.71 to 1.00, except for UDP98-407 loci. The average of observed heterozygosity in Lithuanian-origin cultivars (LT origin-Plum group) and referenced cultivars (R-Plum) is 0.88 and 0.91, respectively. In the LT origin-Plum group, the H_o_ ranged from 0.71–1.00. The highest heterozygosity (1.00) was observed in loci BPPCT040, BPPCT034, BPPCT039, BPPCT007 and CPSCT026. The results are similar in the R-plum group, and two other loci, BPPCT014 and PacA33, show high heterozygosity (1.00). The lowest heterozygosity value (0.33) was observed in the R-Plum group’s locus UDP98-407. However, in the LT origin-Plum group, this locus shows a much higher heterozygosity value (0.71).

According to the PIC value, the most informative primer for the LT origin-Plum group is BPPCT007, and for the R-Plum group—UDP96-005, with the PIC value of 0.374 and 0.414, respectively. The least informative primers for the LT origin-Plum group are BPPCT014 (0.243) and UDP96-005 (0.240). For the R-Plum group, all loci are informative, with an average PIC value of 0.363.

To find the minimal set of primers for genetic diversity, a phylogenetic tree with the most informative primer according to PIC value was constructed. This process was continued by adding primers according to their informativeness (PIC value) until all genotypes were separated. The minimal set for the Lithuanian-origin plum genotype’s genetic diversity analysis consists of two highly informative SSR primers, BPPCT007 and CPSCT026, with PIC values of 0.374 and 0.280, respectively.

The allele frequencies (*p_i_*) of the 14 Lithuanian-origin (LT origin-Plum group) and six reference cultivars (R-Plum group) ranged from 5% to 85% (Table 2). Of the 157 polymorphic alleles, 79 (50.3%) alleles were common for the Lithuanian-origin and reference cultivars, 62 (39.5%) were unique alleles in the Lithuanian-origin cultivars, and only 16 (10.2%) were unique alleles in the reference cultivars. The alleles with frequency values of *p_i_* ≤ 10% were detected as rare. Of the alleles common to both groups of analyzed cultivars, only 10.1% (8 alleles) were classified as rare. In three loci: BPPCT040, BPPCT039, and UDP98-407, rare alleles were absent at all. Most unique alleles in Lithuanian-origin cultivars were rare, 80.6% (50 alleles) and observed in all nine loci. All 16 unique alleles in reference cultivars were rare and observed at BPPCT034, BPPCT039, UDP98-407, PacA33, CPSCT026 and UDP96-005 loci.

In Lithuanian-origin cultivars (LT origin-Plum group), homozygosity was observed at four loci (BPPCT014, UDP98-407, Pa-cA33, and UDP96-005) (Table S1). The most homozygous Lithuanian plum cultivars were ‘Gyne’ (homozygous at four loci), ‘Aleksona’ (homozygous at three loci), and ‘Kauno vengrine’ (homozygous at two loci). The least homozygous (homozygous at one locus) were eight Lithuanian-origin cultivars: ‘Orija’, ‘Zalioji renklode’, ‘Vilniaus Vengrine’, ‘Altano renklode’, ‘Jure’, ‘Katra’, ‘Rype’ and ‘Skalve’. Only heterozygotic loci were observed in three Lithuanian-origin cultivars (‘Vietine geltonoji’, ‘Alge’, and ‘Staro Vengrine’).

To evaluate the polymorphism of 39 European plum hybrids and six parental forms, 191 and 109 alleles, respectively, were observed (Table 3). In hybrids, the allele number ranged from 14 to 26, with an average of 21.22. In the parental forms of hybrids, the allele number range was much narrower, from 8 to 17, with an average of 12.11. In hybrids, the highest number of alleles (26) was observed in the BPPCT040 and PacA33 loci, which were the most informative according to allele number. Additionally, the BPPCT040 locus was the most informative for the parental forms of the hybrids, with the highest number (17) of alleles observed. However, the PacA33 loci had the lowest number of alleles in the parental forms of the hybrids.

The allelic size range in hybrids and their parental forms was the same in three loci: BPPCT007, CPSCT026, and UDP96-005. In hybrids, the allele size range in five loci (BPPCT040, BPPCT034, BPPCT014, UDP98-407, PacA33) was wider than in their parental forms. Only in one locus, BPPCT039, the allelic size range was wider in the parental forms of the hybrids (Table 3).

The average observed heterozygosity in hybrids was higher than in their parental forms (0.93 and 0.91, respectively). At loci BPPCT040, BPPCT034, BPPCT039, BPPCT00, and CPSCT026, the observed heterozygosity in hybrids and their parental forms was 1.00. In the parental forms of hybrids, heterozygosity was high in locus BPPCT014 (1.00); however, in hybrids, the heterozygosity was lower (0.95) in this locus. The lowest heterozygosity value was observed in UDP98-407 in the hybrid parental forms (0.5).

According to the PIC value, primer informativeness for the parental forms of hybrids varies from 0.284 to 0.363, with an average of 0.342 in all analyzed loci. However, for the hybrids, the PIC value was lower (from 0.157 to 0.279, with an average of 0.223).

**Figure 1:**
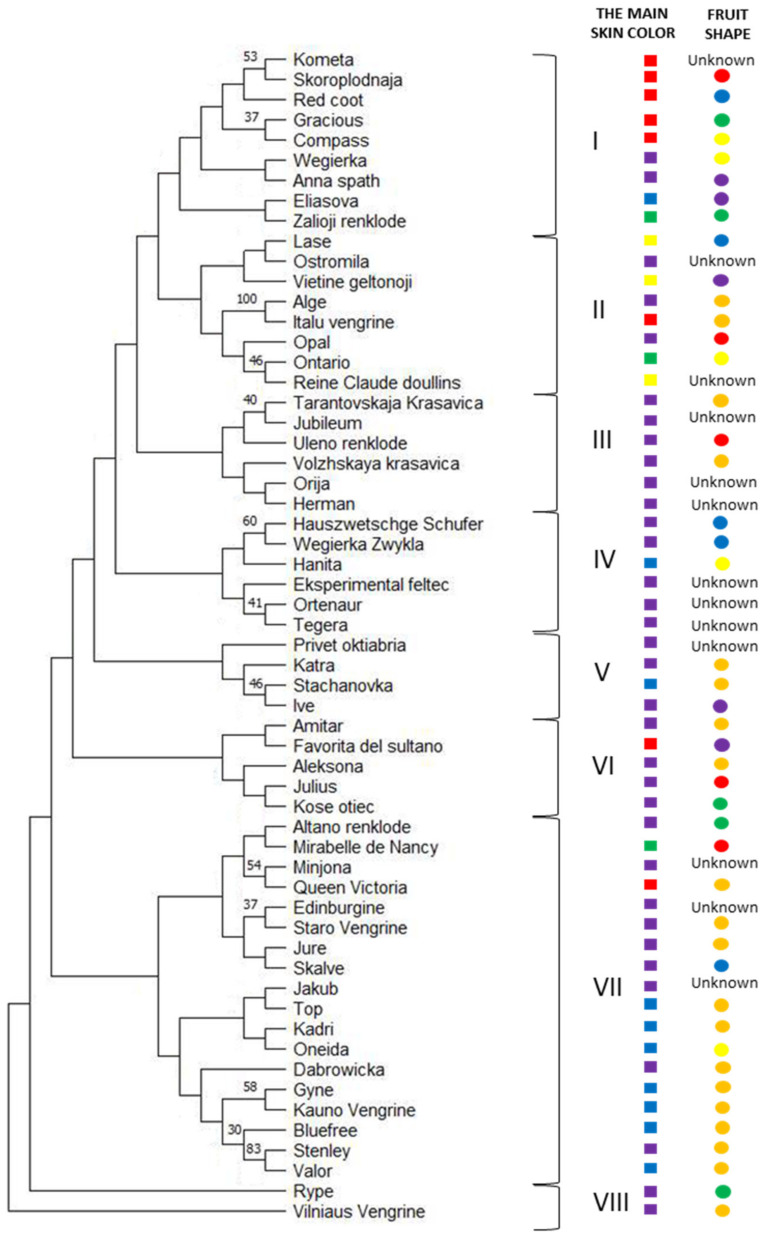
Phylogenetic tree of European plum cultivars growing in Lithuania using 9 SSR primers data (values above branches indicate bootstrap values ≥ 30%). The clusters of the phylogenetic tree are marked with I–VIII numbers (see in the text). On the right side of the dendrogram: the main color of the skin is marked in colored circles matching the skin color of the fruit: green, yellow, orange, red, purple and blue. The fruit shape is marked in colored circles: Blue—elongated; Orange—elliptical; Yellow—oval; Red—round; Green—flat; Purple—oval reverse; Unknown—the shape of the fruit was not evaluated.

The phylogenetic tree was constructed to evaluate genetic diversity and relations between data from 9 SSR loci and morphological traits of 58 European plum cultivars (Figure 1). European plum cultivars grown in Lithuania were grouped into eight different branches. Lithuanian-origin cultivars were distributed among all phylogenetic tree clusters except in cluster IV.

The first cluster (I) consists of nine cultivars, including the international reference cultivar ‘Anna spath’ and the Lithuanian cultivar ‘Zalioji renklode’. The second cluster (II) consists of seven European cultivars and one cultivar from the USA. Two Lithuanian cultivars, ‘Vietine geltonoji’ and ‘Alge’, belong to this group. The cultivar ‘Alge’ was grouped with the Italian cultivar ‘Italu vengrine’ with significant bootstrap value. This cluster also includes France’s international reference cultivar ‘Reine Cloude doullins’. The third cluster (III) consists of six cultivars from Russia and Europe, including a Lithuanian cultivar, ‘Orija’. In the fifth cluster (V), four cultivars from Russia, Lithuania and Sweden can be found. The Lithuanian cultivar ‘Katra’ was closest to the Russian cultivars ‘Privet oktiabra’ and ‘Stachanovka’. The sixth cluster (VI) consists of five European cultivars and one Russian cultivar. The Lithuanian cultivar ‘Aleksona’ was the most similar to ‘Julius’ from Estonia.

The seventh cluster (VII) was the largest group and consisted of two subgroups. For the first subgroup, four Lithuanian cultivars ‘Altano renklode’, ‘Staro Vengrine’, ‘Jure’, ‘Skalve’, and an international reference cultivar ‘Mirabelle de Nancy’ from Trance, a Latvian cultivar and two cultivars from the United Kingdom belonged. The second subgroup comprises ten cultivars from Russia, Europe, the USA and Canada, including two international reference cultivars, ‘Stenley’ and ‘Valor’, with significant bootstrap value (83%).

Cultivars were grouped according to geographical location only in two clusters. The fourth cluster (IV) consisted of cultivars only from Europe, including the international reference cultivar ‘Hanita’. The eighth cluster (VIII) consists of two Lithuanian cultivars, ‘Rype’ and ‘Vilniaus Vengrine’.

Morphological data of European plum cultivars fruits is provided in Figure 1. The cultivars with purple skin color were grouped into third and eighth clusters. Three clusters (IV, V and VI) consist of cultivars with purple skin color except for one cultivar with different skin color in each group: in the fourth and fifth groups, ‘Hanita’ and ‘Stachanovka’ cultivars with blue skin color, and in the sixth group—Italian cultivar ‘Favorita del sultano’ with red skin color. Cultivars with yellow skin color can be found only in the second group (II), and cultivars with red skin color are grouped mainly into the first cluster of the phylogenetic tree. Cultivars with blue skin color are mostly grouped into the seventh cluster. Cultivars grouped in the phylogenetic tree with strong bootstrap support have the same fruit shape: ‘Gyne’, ‘Kauno vengrine’, ‘Bluefree’, ‘Stenley’, ‘Valor’, ‘Alge’ and ‘Italu vengrine’—elliptical fruit shape, ‘Hauszwetschge Schufer’ and ‘Wegierka Zvykla’—elongated fruit shape (Figure 1).

Two cultivars were grouped according to the pedigree. Cultivars ‘Stenley’ and ‘Valor’ are grouped in the same cluster with significant bootstrap. These two cultivars, ‘Stenley’ (‘d‘Agen’ × ‘Grand Duke’) from the USA and ‘Valor’ (‘Imperial Epineuse’ × ‘Grand Duke’) from Canada, might be grouped together because of the same parents from ‘Grand Duke’.

**Figure 2:**
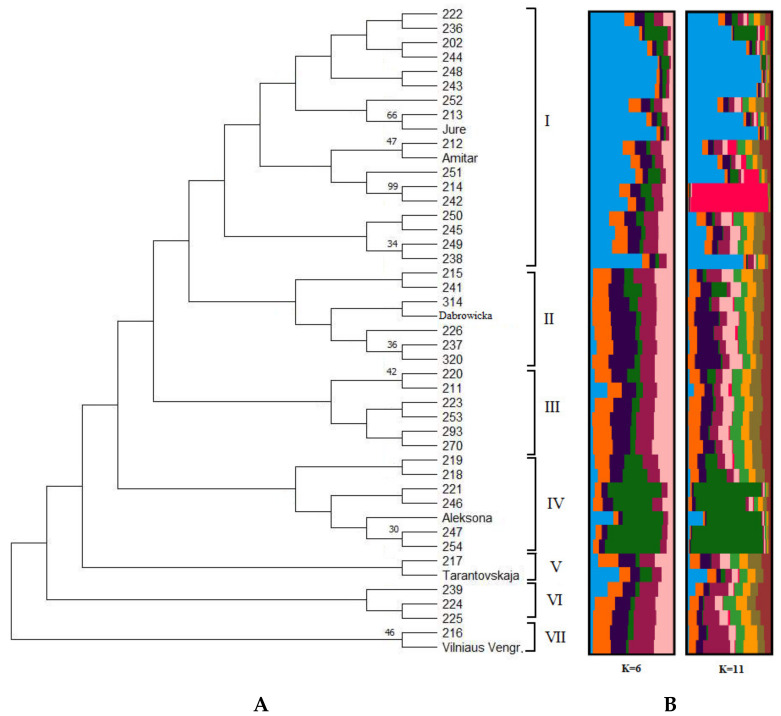
Genetic structure of European plum hybrids and their parental forms growing in Lithuania. (**A**) Phylogenetic tree of European plum genotypes (values above branches indicates bootstrap values ≥ 30%). The clusters of the phylogenetic tree are marked with I–VII numbers. (**B**) A bar plot of the results of the Bayesian clustering analysis on plum species genotypes, revealed by the STRUCTURE program, with K = 6 and K = 11.

The phylogenetic tree was constructed for hybrids and their parental forms. All genotypes were grouped into seven clusters according to pedigree (Figure 2A). The first cluster (I) is the largest in the phylogenetic tree, containing 18 genotypes of hybrids with ‘Jure’ and ‘Amitar’ cultivars as parental forms. The second cluster (II) consists of the ‘Dabrowicka’ cultivar and hybrids of the ‘Dabrowicka’ pedigree. The second largest cluster (IV) consists of seven hybrids genotypes with ‘Aleksona’ and ‘Harmonija’ cultivars as their parental forms. The sixth (VI) and seventh (VII) clusters consist of hybrids with ‘Vilniaus Vengrine’ as one of the parental forms. However, some hybrids (Vilniaus Vengrine × Jure − 238 (cluster I), 239 (cluster VI), 216 (cluster VII); Aleksona × Harmonija − 220 (cluster III), 221, 246, 247, 254 (cluster IV) and free pollination of Cacanska najbolja − 211, 293 (Cluster III), 219,218 (Cluster IV), 217 (Cluster V)) with the same parental forms belonged to different clusters.

**Figure 3:**
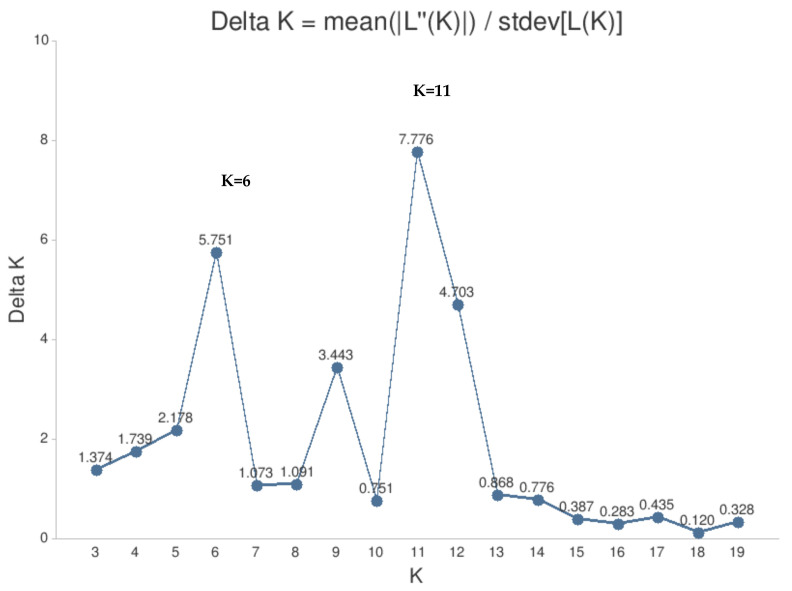
Estimation of the most probable K value for analyzed plum hybrid and their parental forms, based on the Evanno et al. [31] method.

Bayesian clustering analysis was performed with STRUCTURE software, based on nine SSR loci for all 45 genotypes of hybrids and parental forms. A bar plot of the results is provided on the right side of the phylogenetic tree (Figure 2B). Within the studied genotypes, the two highest values of K = 6 and K = 11 were obtained (Figure 3). In both cases, the first (blue) cluster includes the first group (I) of the phylogenetic tree and the green cluster—the fourth (IV) group of the phylogenetic tree. In the case where eleven clusters were defined (K = 11), a separate group of two genotypes (with strong bootstrap support and the same pedigree) was distinguished into the phylogenetic tree’s first group.

## 3. Discussion

The populations of plant species on the border of the spread are more unique than populations in the central part of the areal [30]. Higher heterozygosity values and the presence of rare or unique alleles or higher polymorphism express the uniqueness. Developing new or extant unique alleles in such border populations is mainly related to survival or adaptation to environmental conditions. It is essential for conserving the genetic diversity of genotypes. Lithuania is in the northern part of the distribution area of plums. The main traits for plum adaption are winter harshness and occasional spring frosts during plum flowering. However, global warming and climate change are new challenges for breeding plums and related species in many countries, so the available cultivar’s growth needs improvement. It is essential to know the available genetic resources to create new plum cultivars with desired ecological and biological adaptive characteristics [32,33].

For plant species, molecular markers provide tools for genetic diversity analysis and genome structure studies. Microsatellites (SSR) are polymorphic and codominant, meaning they can be beneficial for cultivar identification, phylogenetic studies and species fingerprinting [34].

In this study, the genetic diversity of 68 European plum cultivars grown in Lithuania was analyzed, including six foreign reference cultivars: ‘Anna spath’ (Germany), ‘Hanita’ (Germany), ‘Mirabelle de Nancy’ (France), ‘Reine Claude doullins’ (France), ‘Stenley’ (United States), ‘Valor’ (Canada), 14 cultivars of Lithuanian origin and 48 foreign cultivars. SSR analysis of genotypes showed a high level of genetic diversity in plums.

The genotyping data of referenced cultivars analyzed in this research agrees with the results of Nybom et al. [3]. However, a 1–4 bp shift is noticeable in the allele size range. The observed heterozygosity of reference genotypes in this study coincides with Nybom et al. [3] results, with a slightly higher value (0.91) in this study compared to Nybom et al. [3] results (0.895). The samples of reference cultivars in Nybom et al. [3] research were collected in Croatia, the central European part, where the heterozygosity of species is lower than in northern Europe [35], so it could indicate a possible adaptation of cultivars to northern conditions.

In this study, the mean value of alleles per locus in all analyzed genotypes was lower (24.33) compared to the study of genotypes sampled across European countries like Great Britain, Estonia, Latvia, Norway, Sweden, Italy and Greece [6] (in average 29.3) and to France accessions (29 alleles) [18]. However, the average value of allele number per locus in this study was higher compared to the studies in Spain [11], Nordic [36], Germany [28], Hungary [19] and Croatia [17]. The mean value of alleles per locus of this research is the most similar to Urrestarazu et al. [11] results despite the higher number of genotype accessions (166) and the number of used SSR markers. Such differences could be due to various accessions and evaluations of different pomological plum groups or geographical locations.

The highest number of alleles in all analyzed genotypes was observed with the PacA33 primer pair and the lowest number with the BPPCT014 primer pair. The opposite results of the BPPCT014 primer pair were observed in Gaši et al. [6], Sehic et al. [36] and Manco et al. [20] research—where the BPPCT014 primer pair generated the highest number of alleles. This opposition of BPPCT014 primer pair allele number may have arisen because of allele ranges analyzed in the research. In this research, the allele range of the BPPCT014 primer pair was 185–225 bp, while in another research—it was 186–289 [6]. The calculation of allelic number, frequency, and other related parameters might be affected by the polyploidy level of the European plum, while the rare alleles are over-represented [20].

According to SSR analysis, the most informative primer pairs for Lithuanian-origin cultivars (LT origin-Plum group) were BPPCT040—with the highest number of alleles and BPPCT007 and CPSCT026 with the highest value of polymorphism information content (PIC).

A wide range of unique alleles was observed among the analyzed Lithuanian genotypes, which shows the genetic individuality of the population. In Lithuanian-origin cultivars, 62 (39.5%) alleles were observed as unique, indicating genetic diversity, which is essential for adaptation to strict ecological conditions of the northern part of the species’ habitat [30]. This genetic plant material is essential to use in breeding to increase the genetic variability of plum species [19]. From the polymorphic alleles of analyzed genotypes, 16 (10.2%) unique alleles were found in referenced cultivars growing in Lithuania. Those alleles are lost over time while not linked to the traits needed for cultivars to survive under specific ecological conditions.

The cluster analysis of studied cultivars shows that foreign and domestic cultivars do not form separate groups according to geographical location; the same tendency of results distribution in the phylogenetic tree clusters is shown in other research works [6,25]. Genotype differentiation linked to geographical origin was analyzed by Gaši et al. [6] using AMOVA, and only 0.7% of the total variation was linked to geographical location.

In the cluster analysis of hybrids, all hybrids clustered with their parental forms according to their pedigree. The same clustering tendency was noted in this study with ‘Stenley’ and ‘Valor’ cultivars and in Makovics-Zxohar et al. [19] research.

In this research, morphological traits did not have significant implications for SSR results; only the skin color of the plum fruit had an impact on the cultivar’s genetic clustering results. In most research, no significant correlation was observed between morphological traits and molecular data [17,18]. However, pomological assignment is important for explaining European plum genotype clustering [6].

## 4. Materials and Methods

In total, 107 European plum (*Prunus domestica*) cultivars (Table S2) and hybrids (Table S3) were analyzed in this study: 39 hybrids and 68 European plum cultivars, including six internationally referenced cultivars (R-Plum group), 14 Lithuanian-origin cultivars (LT origin-Plum group), 48 accessions from Lithuania genetic resources collection of plum (LT-Plum group). For the genetic analysis of hybrids, six parental forms (‘Aleksona’, ‘Tarantovskaja krasavica’, ‘Vilniaus Vengrine’, ‘Jure’, ‘Dabrowicka’ and ‘Amitar’) genotypes SSR data were analyzed (Table S3). Other parental forms, provided in Table S3, were excluded from the genetic structure analysis (SSR data of these cultivars was not analyzed).

For 58 European plum cultivars, the morphological features—the main skin color and the fruit shape, were visually evaluated in the Lithuanian Research Centre for Agriculture and Forestry institute of horticulture orchards in 2018–2022, when the fruit reached ripening fruit and seed principal growth stage (BBCH 86–89).

The leaves were collected from one-year shots in the spring from a single plum tree for each of analyzing plum cultivars at the Lithuanian Research Centre for Agriculture and Forestry institute of horticulture in 2021. Leaves were frozen with nitrogen and kept at −70 °C until further analysis. DNA was extracted using a modified [30] Cetyltrimethyl ammonium bromide (CTAB) method [37]. For SSR analysis, the standard set of nine SSR primer pairs for plums, according to ECPGR recommendations, was used [3] (Table 4). PCR amplifications were performed with 10 µL a total volume of the reaction mixture, consisting of (300 ng/µL) DNA, 0.2 mM of each primer, 25 mM of MgCl, 2 mM dNTP, 10× buffer, 10 mM DTT, 1% PVP, 500 U Taq DNA polymerase (Thermo Scientific, Wilmington, DE, USA). Each forward primer was labeled with 6-FAM (Blue), HEX (Green) or ATTO550 (Yellow) fluorescent dye (Applied Biosystems, Foster City, CA, USA). Amplification of DNA fragments was performed in a thermocycler (Eppendorf, Germany) with the conditions as follows: initial denaturation for 10 min at 94 °C, followed by seven cycles with touchdown procedure at primer annealing step: 30 s at 94 °C, 45 s at X °C (−1°C in each cycle), 1 min at 72 °C; and 25 cycles—30 s at 94 °C, 45 s at Y °C, 1 min at 72 °C, the final fragment synthesis carried out for 10 min at 72 °C, where Y is the appropriate annealing of primer (Table 4) and X = Y + 7 the initial temperature for each primer by touchdown procedure. Capillary electrophoresis was performed with ABI 3130 Genetic Analyzer (Applied Biosystems, Foster City, CA, USA) using standard (GeneScan 500LIZ).

The observed heterozygosity, frequency of alleles, and polymorphism information content (PIC) were calculated for each of nine SSR primer pairs according to Roldán-Ruiz et al. [41]. The informativeness of SSR loci was expressed by PIC value [42]. The phylogenetic tree was constructed using MEGA X (Molecular Evolutionary Genetics Analysis) software [43] with an Unweighted Pair Group Method with Arithmetic mean (UPGMA) [44]. The evolutionary distances were computed using the Maximum Composite Likelihood method [45]. The bootstrap test was performed with 1000 replicates. Numbers above the phylogenetic tree branches show bootstrap values (more than 30%) [46].

The genetic structure of hybrids and their parental cultivars was determined using the Bayesian model-based clustering method with Structure v. 2.2.3 software [47]. Absent alleles were treated as missing data and marked -9 for individuals with less than six allelic variants per locus. The program applies the results according to K value—assumed genetic groups, each characterized by a subset of allele frequencies determined in the data [48]. Ten independent runs were conducted for each K. K reconstructed panmictic populations (RPPs) were computed on individuals, testing K (log-likelihood) = 1–20 for all samples. A burn-in period of 200,000 and 500,000 iterations was applied. Structure Harvester version 0.6.1 [49], which implements the Evanno et al. method [31], was used to estimate K values for the analyzed data.

## 5. Conclusions

The application of microsatellite markers to European plum cultivars growing in Lithuania enabled the characterization and identification of genotypes and highlighted the uniqueness of analyzed cultivars. Lithuanian-origin European plum cultivars have various unique alleles essential for plant breeding under exceptional northern climate conditions. ECPGR recommended primer set for SSR analysis of European plum cultivars is appropriate for genetic diversity analysis of Lithuanian-origin cultivars (LT origin-Plum group). According to the highest number of alleles BPPCT040 primer pair is the most informative, and the highest polymorphism information content was determined with BPPCT007 primer pair. According to the PIC value, the least informative primer pair for Lithuanian cultivars (LT origin-Plum group) fingerprinting was UDP96-005. The minimal set for the plum genotype’s genetic diversity analysis consists of two highly informative SSR primers, BPPCT007 and CPSCT026, with PIC values of 0.374 and 0.280, respectively. However, it would be beneficial to use a complete primer set for molecular fingerprinting and genetic diversity evaluation of European plum. In the phylogenetic analysis, cultivars were grouped according to the pedigree. However, geographical location and morphological traits did not significantly affect SSR results.

## Figures and Tables

**Table 1:** Comparison of microsatellite loci characteristics between Lithuanian origin cultivars (LT origin-Plum group) and reference cultivars (R-Plum).

		Lithuanian-Origin Cultivars (LT Origin-Plum Group)					Reference Cultivars (R-Plum)				
	Marker Name	Allele Size Range (bp)	Allele Number	H_o_ ^ 1^	PIC ^2^	Max. Number of Alleles in Genotype	Allele Size Range (bp)	Allele Number	H_o_ ^ 1^	PIC ^2^	Max. Number of Alleles in Genotype
1.	BPPCT040	115–155	21	1.00	0.274	6	116–147	8	1.00	0.410	5
2.	BPPCT034	216–258	17	1.00	0.256	6	216–258	13	1.00	0.308	4
3.	BPPCT039	126–171	15	1.00	0.286	5	126–177	13	1.00	0.368	5
4.	BPPCT014	185–225	12	0.71	0.243	5	185–225	9	1.00	0.358	5
5.	UDP98-407	168–197	11	0.71	0.258	3	164–194	6	0.33	0.343	3
6.	PacA33	175–213	13	0.79	0.265	6	168–210	11	1.00	0.333	4
7.	BPPCT007	124–161	14	1.00	0.374	6	124–149	11	1.00	0.369	6
8.	CPSCT026	166–213	18	1.00	0.280	6	166–210	13	1.00	0.368	3
9.	UDP96-005	96–154	19	0.71	0.240	6	104–152	11	0.83	0.414	5
Average			15.67	0.88	0.275	5.44		10.56	0.91	0.363	4.44

^1^ H_o_—observed heterozygosity; ^2^ PIC—polymorphism information content.

**Table 2:** Alleles and their frequencies in Lithuanian-origin cultivars (LT origin-Plum group)) and reference (R-Plum group) plum cultivars.

Loci	Common Allele Lengths in bp (Frequencies in %)	Unique Allele Lengths in bp (Frequencies in %) *	
		Lithuanian Origin Cultivars	Reference Cultivars
BPPCT040	116 (20); 120 (25); 124 (35); 126 (50); 128 (50); 134 (30); 145 (25); 147 (40)	**115 (5); 118 (5); 119 (5); 131 (5); 136 (10); 138 (5); 140 (10); 141 (5); 144 (10); 149 (5); 150 (5);** 153 (15); 155 (15)	-
BPPCT034	216 (75); 226 (25); **229 (10);** 235 (20); 236 (15); **238 (10);** 241 (55); 243 (20); 250 (15); 258 (15)	**222 (10); 227 (5); 232 (5);** 234 (35); **237 (5); 246 (10); 247 (5)**	**225 (5); 249 (5); 256 (5)**
BPPCT039	126 (35); 129 (20); 131(15); 132 (30); 136 (25); 143 (25); 145 (15); 150 (15); 153 (70); 171 (25)	**128 (10); 130 (10); 141 (10); 163 (5); 167 (10)**	**139 (5); 159 (5); 177 (10)**
BPPCT014	185 (85); 202 (20); 204 (45); 214 (15); 215 (20); 216 (15); **218 (10); 223 (10);** 225 (25)	203 (15); **208 (5); 221 (5)**	-
UDP98-407	181 (30); 187 (25); 194 (20)	**168 (10); 172 (5); 179 (25); 186 (5); 189 (5);** 191 (15); 193 (15); **197 (5)**	**164 (5); 177 (5); 185 (5)**
PacA33	175 (50); 177 (15); 185 (30); 193 (15); 194 (30); 196 (10); **209 (10)**	**179 (10);** 183 (20); 191 (15); **192 (5); 202 (5); 213 (10)**	**168 (5); 198 (5); 206 (5); 210 (5)**
BPPCT007	124 (40); 126 (20); 128 (40); 130 (55); 134 (60); 136 (20); 138 (45); 140 (50); 142 (20); **144 (10);** 149 (60)	**134 (10); 146 (10);** 151 (30); **161 (5)**	-
CPSCT026	166 (55); 175 (25); 183 (25); **188 (10);** 189 (80); 193 (35); 196 (25); 199 (35); 200 (20); 202 (55); 204 (25); 210 (15)	**173 (5);** 195 (15); 197 (15); **208 (5); 211 (5); 213 (5)**	**185 (5)**
UDP96-005	104 (25); **105 (10);** 107 (20); 112 (30); 115 (20); 125 (15); 137 (15); 148 (35); 152 (30)	**96 (10); 120 (10); 124 (10); 128 (5); 130 (10); 132 (5); 134 (5); 139 (10); 142 (10);** 154 (15)	**127 (5); 150 (10)**
No. of alleles	79 (50.3%)	62 (39,5%)	16 (10,2%)
No. of rare alleles	8 (10,1%)	50 (80,6%)	16 (100%)

* Values in bold are rare alleles (frequency *p_i_* ≤ 10%), Unique allele—the genetic individuality of a plant population [30].

**Table 3:** Comparison of microsatellite loci characteristics between hybrids and their parental form.

		Hybrids				Parental Forms of Hybrids			
	Marker Name	Allele Size Range (bp)	Allele Number	H_o_ ^1^	PIC ^2^	Allele Size Range (bp)	Allele Number	H_o_ ^1^	PIC ^2^
1.	BPPCT040	115–155	26	1.00	0.253	117–155	17	1.00	0.333
2.	BPPCT034	216–256	22	1.00	0.221	216–250	15	1.00	0.352
3.	BPPCT039	124–177	23	1.00	0.251	126–177	13	1.00	0.363
4.	BPPCT014	185–225	14	0.95	0.225	185–221	9	1.00	0.284
5.	UDP98-407	164–225	17	0.80	0.179	164–198	9	0.50	0.358
6.	PacA33	168–221	26	0.82	0.157	168–194	8	0.83	0.347
7.	BPPCT007	124–161	18	1.00	0.279	124–161	12	1.00	0.352
8.	CPSCT026	166–211	20	1.00	0.260	166–211	13	1.00	0.333
9.	UDP96-005	96–154	25	0.77	0.179	96–154	13	0.83	0.355
Average			21.22	0.93	0.223		12.11	0.91	0.342

^1^ H_o_—observed heterozygosity; ^2^ PIC—polymorphism information content.

**Table 4:** SSR primer pairs.

Locus Name	DNA Sequence	Dye	Annealing Temp. °C	Reference
UDP 98-407	5′-AGCGGCAGGCTAAATATCAA-3′ 5′-AATCGCCGATCAAAGCAAC-3′	HEX	58	Cipriani et al. [38]
Pac A 33	5′-TCAGTCTCATCCTGCATACG-3′ 5′-CATGTGGCTCAAGGATCAAA-3′	ATTO550	58	Decroocq et al. [39]
CPSCT 026	5′-TCTCACACGCTTTCGTCAAC-3′ 5′-AAAAAGCCAAAAGGGGTTGT-3′	6-FAM	46	Mnejja et al. [22]
BPPCT 040	5′-ATGAGGACGTGTCTGAATGG-3′ 5′-AGCCAAACCCCTCTTATACG-3′	6-FAM	58	Dirlewanger et al. [40]
BPPCT 007	5′-TCATTGCTCGTCATCAGC-3′ 5′-CAGATTTCTGAAGTTAGCGGTA-3′	HEX	60	Dirlewanger et al. [40]
BPPCT 034	5′-CTACCTGAAATAAGCAGAGCC AT-3′ 5′-CAATGGAGAATGGGGTGC-3′	6-FAM	56	Dirlewanger et al. [40]
UDP 96-005	5′-GTAACGCTCGCTACCACAAA-3′ 5′-CCTGCATATCACCACCCAG-3′	HEX	56	Cipriani et al. [38]
BPPCT 039	5′-ATTACGTACCCTAAAGCTTCTGC-3′ 5′-GATGTCATGAAGATTGGAGAGG-3′	HEX	58	Dirlewanger et al. [40]
BPPCT 014	5′-TTGTCTGCCTCTCATCTTAACC-3′ 5′-CATCGCAGAGAACTGAGAGC-3′	6-FAM	58	Dirlewanger et al. [40]

## Data Availability

Data are contained within the article or Supplementary Materials.

## Supplementary Materials

The following supporting information can be downloaded at: https://www.mdpi.com/article/10.3390/plants12071538/s1. Table S1: Molecular profiles for all European plum cultivars (the length of detected alleles are provided in bp); Tabel S2: European plum cultivars origin; Table S3: Parental forms of hybrids.

## Appendix A

**Table A1:** Primer informativeness of all analyzed European plum cultivars (R-plum, LT origin-Plum group and LT-Plum groups) and hybrids growing in Lithuania.

	Marker Name	Allele Size Range (bp)	Allele Number	H_o_ ^1^	PIC ^2^
1.	BPPCT040	115–155	27	0.991	0.232
2.	BPPCT034	216–258	24	0.981	0.213
3.	BPPCT039	124–177	24	1	0.239
4.	BPPCT014	185–225	18	0.869	0.182
5.	UDP98-407	164–233	24	0.766	0.139
6.	PacA33	168–223	30	0.832	0.136
7.	BPPCT007	124–161	20	0.832	0.282
8.	CPSCT026	166–213	24	0.991	0.235
9.	UDP96-005	96–154	28	0.757	0.162
Average			24.33	0.891	0.203

^1^ H_o_—observed heterozygosity; ^2^ PIC—polymorphism information content.
