# Supplementary material for: Fingerprinting of Plum (Prunus domestica) Genotypes in Lithuania Using SSR Markers
Source: Plants (Basel). 2023 Apr 3;12(7):1538. doi: 10.3390/plants12071538 (PMC10097231; doi:10.3390/plants12071538)
Supplement: Supplementary file 1 [file plants-12-01538-s001.zip › Supplements 2.pdf]

Tabel S2. European plum cultivars origin

No.	Cultivar	Country of origin
**Accessions from Lithuania genetic resources collection of plum (LT-Plum group)**		
1.	Amitar	Estonian
2.	Bluefree	USA
3.	Cacak Beauty	Yugoslavia
4.	Compass	USA
5.	Dabrowicka	Poland
6.	Edinburgine	UK
7.	Eksperimental feltec	Sweden
8.	Eliasova	Czechoslovakia
9.	Favorita del sultano	Italy
10.	Gracious	USA
11.	Hauszwetschge Schufer	Germany
12.	Herman	Sweeden
13.	Italu vengrine	Italy
14.	Ive	Sweeden
15.	Jakub	Russia
16.	Jubileum	Sweeden
17.	Julius	Estonia
18.	Kadri	Estonia
19.	Kometa	Russia
20.	Kose otiec	Russia
21.	Lase	Latvia
22.	Minjona	Latvia
23.	Oneida	Italy
24.	Ontario	USA
25.	Opal	Sweeden
26.	Ortenaur	Unknown
27.	Ostromila	Bulgaria
28.	Privet oktiabria	Russia
29.	Queen Victoria	UK
30.	Red coot	USA
31.	Skoroplodnaja	Russia
32.	Stachanovka	Russia
33.	Tarantovskaja Krasavica	Russia
34.	Tegera	Germany
35.	Top	Germany
36.	Uleno renklode	France
37.	Unknown cultivar (100-2)	Unknown
38.	Unknown cultivar (1614)	Unknown
39.	Unknown cultivar (2245)	Unknown
40.	Unknown cultivar (SR Geltona)	Unknown
41.	Unknown cultivar from Belarus	Belarus
42.	Unknown cultivar from Kedainiai dist.	Unknown
43.	Unknown cultivar from Siauliai dist.	Unknown
44.	Unknown cultivar from Russia	Russia

45.	Unknown cultivar from Sweeden (Prunus Nr.2)	Sweeden
46.	Volzhskaya krasavica	Russia
47.	Wegierka	Poland
48.	Wegierka Zwykla	Poland
	**Reference cultivars (R-Plum group)**	
49.	Anna spath	Germany
50.	Hanita	Germany
51.	Mirabelle de Nancy	France
52.	Reine Claude doullins	France
53.	Stenley	US
54.	Valor	Canada
**Lithuanian-origin cultivars (LT origin-Plum group)**		
55.	Vietine geltonoji	Lithuania
56.	Orija	Lithuania
57.	Zalioji renklode	Lithuania
58.	Alge	Lithuania
59.	Vilniaus Vengrine	Lithuania
60.	Aleksona	Lithuania
61.	Altano renklode	Lithuania
62.	Gyne	Lithuania
63.	Jure	Lithuania
64.	Katra	Lithuania
65.	Kauno vengrine	Lithuania
66.	Rype	Lithuania
67.	Skalve	Lithuania
68.	Staro Vengrine	Lithuania
