# Supplementary material for: Fingerprinting of Plum (Prunus domestica) Genotypes in Lithuania Using SSR Markers
Source: Plants (Basel). 2023 Apr 3;12(7):1538. doi: 10.3390/plants12071538 (PMC10097231; doi:10.3390/plants12071538)
Supplement: Supplementary file 1 [file plants-12-01538-s001.zip › Supplements 3 .pdf]

Table S3. Parental forms of hybrids

No.	Hybrid number	Parental Forms
1.	PL-202	Amitar × Jure
2.	PL-211	Free pollination of Cacanska najbolja
3.	PL-212	Amitar × Jure
4.	PL-213	Amitar × Jure
5.	PL-214	Cacanska najbolja × Jure
6.	PL-215	Vilniaus Vengrine × Harmonija
7.	PL-216	Vilniaus Vengrine × Jure
8.	PL-217	Free pollination of Cacanska najbolja
9.	PL-218	Free pollination of Cacanska najbolja
10.	PL-219	Free pollination of Cacanska najbolja
11.	PL-220	Aleksona × Harmonija
12.	PL-221	Aleksona × Harmonija
13.	PL-222	Aleksona × Jure
14.	PL-223	Harmonija × Jure
15.	PL-224	Free pollination of I-1546
16.	PL-225	Free pollination of I-1546
17.	PL-226	Free pollination of Jojo
18.	PL-236	Aleksona × Jure
19.	PL-237	Dabrowicka × Jure
20.	PL-238	Vilniaus Vengrine × Jure
21.	PL-239	Vilniaus Vengrine × Jure
22.	PL-241	Vilniaus Vengrine × Harmonija
23.	PL-242	Cacanska najbolja × Jure
24.	PL-243	Aleksona × Jure
25.	PL-244	Tarantovskaja krasavica × Jure
26.	PL-245	Dabrowicka × Jure
27.	PL-246	Aleksona × Harmonija
28.	PL-247	Aleksona × Harmonija
29.	PL-248	Aleksona × Jure
30.	PL-249	2134 × Harmonija
31.	PL-250	Harmonija × Jure
32.	PL-251	Aleksona × Jure
33.	PL-252	6002a × Jure
34.	PL-253	Free pollination of I-1546
35.	PL-254	Aleksona × Harmonija
36.	PL-270	Free pollination of Amers
37.	PL-293	Free pollination of Cacanska najbolja
38.	PL-314	Free pollination of Dabrowicka
39.	PL-320	Free pollination of Dabrowicka
